# Impact of the Dual Deletion of the Mitochondrial Sirtuins SIRT3 and SIRT5 on Anti-microbial Host Defenses

**DOI:** 10.3389/fimmu.2019.02341

**Published:** 2019-10-01

**Authors:** Tytti Heinonen, Eleonora Ciarlo, Didier Le Roy, Thierry Roger

**Affiliations:** Infectious Diseases Service, Department of Medicine, Lausanne University Hospital and University of Lausanne, Epalinges, Switzerland

**Keywords:** sirtuin, innate immunity, cytokine, infection, sepsis, metabolism, macrophage, neutrophil

## Abstract

The sirtuins SIRT3 and SIRT5 are the main mitochondrial lysine deacetylase and desuccinylase, respectively. SIRT3 and SIRT5 regulate metabolism and redox homeostasis and have been involved in age-associated metabolic, neurologic and oncologic diseases. We have previously shown that single deficiency in either SIRT3 or SIRT5 had no impact on host defenses in a large panel of preclinical models of sepsis. However, SIRT3 and SIRT5 may compensate each other considering that they share subcellular location and targets. Here, we generated a SIRT3/5 double knockout mouse line. SIRT3/5 deficient mice multiplied and developed without abnormalities. Hematopoiesis and immune cell development were largely unaffected in SIRT3/5 deficient mice. Whole blood, macrophages and neutrophils from SIRT3/5 deficient mice displayed enhanced inflammatory and bactericidal responses. In agreement, SIRT3/5 deficient mice showed somewhat improved resistance to *Listeria monocytogenes* infection. Overall, the double deficiency in SIRT3 and SIRT5 has rather subtle impacts on immune cell development and anti-microbial host defenses unseen in single deficient mice, indicating a certain degree of overlap between SIRT3 and SIRT5. These data support the assumption that therapies directed against mitochondrial sirtuins, at least SIRT3 and SIRT5, should not impair antibacterial host defenses.

## Introduction

The innate immune system plays a central role in host defenses. Innate immune cells among which monocytes/macrophages, granulocytes and dendritic cells (DCs) sense microbial and danger associated molecular patterns (MAMPs/DAMPs) through pattern recognition receptors (PRRs) expressed at the cell surface, in the cytoplasm and in endosomes. The best characterized PRRs belong to the families of Toll-like receptors (TLRs), C-type lectin receptors (CLRs), NOD-like receptors (NLRs), RIG-I-like receptors (RLRs), and cytosolic DNA sensors (1, 2). The binding of MAMPs/DAMPs to PRRs activates intracellular signaling cascades that induce the production of effector molecules involved in inflammation and host defense mechanisms, as well as the resolution of inflammation and tissue repair (3, 4). Immune cells are plastic and adapt their metabolism and responsiveness to their environment to execute their biological functions (5, 6).

Sirtuins belong to the family of so-called histone deacetylases (HDACs) that target lysine posttranscriptional modifications. Classical HDACs (HDAC1-11) are Zn^2+^-dependent, while sirtuins are NAD^+^-dependent lysine deacetylases. Sirtuins are homologs to yeast Sir2 that gained tremendous attention when it was shown to be activated upon caloric restriction and to increase lifespan (7). The mammalian genome encodes for seven sirtuins that target proteins by removing acetyl functional groups, but also acyl, glutaryl, malonyl, and succinyl groups as demonstrated lately (8). The list of targets of sirtuins has increased dramatically over the years, and high throughput proteomics analyses pinpointed to thousands of substrates for sirtuins. Accordingly, sirtuins are involved in the regulation of many biological and pathological processes and in the development of metabolic, neurodegenerative, cardiovascular, and oncologic diseases (9, 10).

SIRT3 and SIRT5 are mainly localized in the mitochondrial matrix, where SIRT3 is the main deacetylase (11) and SIRT5 is the main desuccinylase (12, 13). Of note, SIRT5 also catalyzes lysine demalonylation and deglutarylation (13, 14). SIRT3 promotes glucose and fatty acid metabolism, urea cycle and the activity of the electron transport chain. During caloric restriction, SIRT3 regulates mitochondrial acetylome and multiple metabolic pathways in the liver (15, 16). SIRT3 protects from oxidative stress by activating the reactive oxygen species (ROS) detoxifying enzyme superoxide dismutase 2 (SOD2) and the redox controlling enzyme isocitrate dehydrogenase 2 (IDH2) (17, 18). Similar to SIRT3, SIRT5 activates enzymes involved in ROS detoxification (i.e., SOD1, IDH1, and IDH2), promotes mitochondrial functions and integrity and regulates the urea cycle and other metabolic pathways (14, 19–25). The genetic ablation of SIRT3 or SIRT5 in mice has been associated with increased susceptibility to age-associated diseases including insulin resistance, obesity, neurodegeneration, cardiac dysfunction and fibrosis, while contrasting context-dependent effects have been reported for tumorigenesis (10, 26–30). Deficiencies in SIRT3 or SIRT5 have also been reported to promote colitis, acute lung injury and ischemia reperfusion injury (10, 31–36). Overall, targeting the activity of sirtuins and particularly mitochondrial sirtuins is viewed as an attractive therapeutic strategy to tackle the development of age-related disorders (10, 28–30). Considering that inflammation is an essential component of innate immune defenses, we analyzed the impact of SIRT3 and SIRT5 deficiencies on the response of mice subjected to a broad panel of preclinical models of bacterial and fungal sepsis (37, 38). Neither SIRT3 nor SIRT5 was critical to fight against infections. Additionally, SIRT3^−/−^ mice were not particularly susceptible to cecal ligation and puncture (CLP), a stringent model of sepsis (39, 40). Hence, SIRT3 and SIRT5 appear to have a more prominent influence on chronic metabolic and inflammation-related disorders than on infectious diseases characterized by acute inflammatory reactions.

SIRT3 and SIRT5 share subcellular location and targets, so they might compensate each other in single knockout mice. To bypass this hurdle, we generated a SIRT3/5 deficient mouse line. SIRT3/5^−/−^ mice were fertile and developed without apparent abnormalities. *In vitro* and *in vivo* investigations revealed somewhat enhanced inflammatory and bactericidal responses of whole blood, macrophages, and neutrophils and a moderate improved resistance to *Listeria monocytogenes* in the double knockouts. Altogether SIRT3 and SIRT5 have subtle, redundant roles during antimicrobial host defenses. Overall, therapies directed against mitochondrial sirtuins should not dramatically impact on antimicrobial host defenses.

## Materials and Methods

### Key Resources

See Supplementary Information.

### Ethics Statement

Animal experiments were approved by the Service des Affaires Vétérinaires, Direction Générale de l'Agriculture, de la Viticulture et des Affaires Vétérinaires (DGAV), état de Vaud (Epalinges, Switzerland; authorizations 876.9 and 877.9) and performed according to Swiss and ARRIVE guidelines (http://www.nc3rs.org.uk/arrive-guidelines).

### Mice

C57BL/6J mice were from Charles River Laboratories (Saint-Germain-sur-l'Arbresle, France). SIRT3^−/−^ and SIRT5^−/−^ C57BL/6J mice were described (41, 42) and obtained from Prof. Johan Auwerx, Laboratory for Integrative and Systems Physiology, Ecole Polytechnique Fédérale de Lausanne, Lausanne, Switzerland. SIRT3^−/−^ males were crossed with SIRT5^−/−^ females. Thirty-two SIRT3/5^+/−^ females were crossed with 16 SIRT3/5^+/−^ males. Among the 205 F2 mice, 4 males and 8 females were double knockout mice and used to establish the SIRT3/5^−/−^ mouse line. All mice used in this study were 7–14-week old, housed under specific pathogen-free conditions and exempt of mouse hepatitis virus and murine norovirus. For genotyping purposes, DNA was extracted and analyzed by PCR using the Mouse Direct PCR Kit (Bimake, Houston, TX) and primers pairs described in Supplementary Information.

### Cells and Reagents

Bone marrow (BM) cells were cultured 7 days in IMDM or RPMI 1640 supplemented with 100 IU/ml penicillin, 100 μg/ml streptomycin (Invitrogen, San Diego, CA), 10% heat inactivated fetal bovine serum (Biochrom GmbH, Berlin, DE) and 50 U/ml macrophage colony-stimulating factor (ImmunoTools, Friesoythe, Germany) or 30% L929 cell supernatant to generate BM-derived macrophages (BMDMs) (43, 44). Cells were seeded in half-area 96-well plates (2.5 × 10^4^ cells/well), 96-well plates (2 × 10^5^ cells/well) and 6-well plates (3 × 10^6^ cells/well) without growth factors. Neutrophils were isolated from the bone marrow using the Neutrophil isolation kit (Miltenyi, Bergisch Gladbach, Germany) and plated in 96-well plates (10^5^ cells/well). *Salmonella minnesota* ultra pure lipopolysaccharide (LPS) was from List Biologicals Laboratories (Campbell, CA), Pam_3_CSK_4_ from EMC microcollections GmBH (Tübingen, Germany), and CpG ODN 1826 (CpG) and poly(I:C) from Invivogen (San Diego, CA). Monosodium urate (MSU) crystals were prepared as described (45). *Listeria monocytogenes* 10403 s was grown in brain heart infusion broth (BD Biosciences, Erembodegem, Belgium). Bacteria were washed with 0.9% NaCl and adjusted at 10^10^ cfu/ml. When required, bacteria were heat-inactivated for 2 h at 70°C.

### RNA Analyses

RNA was extracted (RNeasy kit) and reverse transcribed (QuantiTect reverse transcription kit) (Qiagen, Hilden, Germany). PCRs were performed in triplicate with 1.25 μl cDNA, 1.25 μl H_2_O, 0.62 μl primers [Supplementary Information and (46)] and 3.12 μl KAPA SYBR Green Fast (Kapa Biosystems, Wilmington, MA) using a QuantStudio™ 12K Flex system (Life Technologies, Carlsbad, CA). Gene expression was normalized to actin expression.

### Western Blot Analyses

Total and nuclear proteins were extracted, submitted to PAGE and transferred onto membranes as described (47, 48). Membranes were incubated with primary and secondary HRP-coupled antibodies and revealed by chemiluminescence [Supplementary Information and (49)]. Images were recorded with a Fusion Fx system (Vilber Lourmat, Collégien, France). Full length blots are presented in Supplementary Figure S1.

### Flow Cytometry

Single cell suspensions from thymus, spleen and BM were incubated with 2.4 G2 to block Fc receptors and stained with antibodies described in Supplementary Information (50). For hematopoietic stem cells (HSC) and progenitor cells, lineage cocktail contained antibodies directed against CD45R (B220), CD3e, CD11b, CD19, Ly6C/G, Ter119/Ly-76. Data were acquired using an Attune Nxt flow cytometer (ThermoFisher, Waltham, MA) and analyzed using FlowJo version 10.2 (FlowJo LLC, Ashland, OR). Gating strategies are presented in Supplementary Figure S2.

### ROS Measurement

BMDMs were plated in half-area black 96-well plates in RPMI without phenol red (Invitrogen). Cells were incubated for 10 min at 37°C with 5 μM MitoSOX (Thermofisher). Stimuli were added and fluorescence (Ex_510_, Em_580_) recorded using a Synergy plate reader (BioTek, Winooski, VT). Neutrophils in HBSS without calcium and magnesium (ThermoFisher) were incubated for 1 h with 100 nM PMA (Enzo Life Sciences, Farmingdale, NY) and 5 μM MitoSOX during the last 10 min of incubation. ROS were measured by flow cytometry.

### Cytokine Measurement

Cytokines were quantified by ELISA (Supplementary Information) or Luminex using a custom ProCarta kit (ENA-78/CXCL5, G-CSF, IFNγ, IL-1α, IL-1β, IL-3, IL-6, IL-10, IL-12p40, IL-17A, IL-18, IP-10/CXCL10, KC/CXCL1, MCP-1/CCL2, MIP-1α/CCL3, MIP-2/CXCL2, TNF) (Invitrogen, Carlsbad, CA) and a bioplex 200 system (Bio-Rad, Hercules, CA) (51).

### Metabolic Activity

The metabolic activity of BMDMs was measured using the XF Cell Mito Stress, Glycolysis Stress and Mito Fuel Flex Test Kits on a 96-well format Seahorse XFe^®^ system (Agilent Technologies, Santa Clara, CA) (46).

### Neutrophil Killing and NETosis Assays

Neutrophils were incubated with live *L. monocytogenes* for 1 h in RPMI medium. Serial dilutions of reaction mixtures were plated on blood agar plates (BD Biosciences). Twenty-four hours later, colonies were enumerated. To measure NETosis, neutrophils were incubated for 3 h with 100 nM PMA and 5 μM of the cell impermeable dye Sytox green. Fluorescence (Em_504_, Ex_523_) was recorded using the Synergy plate reader.

### *In vivo* Models

Listeriosis was induced by challenging intravenously (i.v.) age and sex-matched mice with a low (7.3 × 10^3^ cfu) or a high (0.9–1 × 10^5^ cfu) inoculum of *L. monocytogenes*. Blood and organs were collected 1–3 days post-infection to quantify bacteria and cytokines and analyze cell populations. A model of endotoxemia was developed by challenging mice intraperitoneally (i.p.) with 10 mg/kg LPS. Body weight loss, severity score and survival were registered at least twice daily (52, 53) by 2–3 operators. The severity score was graded from 0 to 4 based on the mobility, the posture, the appearance and the weight loss of mice (detailed criteria were approved by the Service des Affaires Vétérinaires, DGAV, and are available upon request). Mice were sacrificed when they met a severity score of 4. A mice found dead was assigned a score of 5.

### Statistical Analyses

Groups were compared by variance analysis followed by two-tailed unpaired Student's *t-*test or a Mann-Whitney test when appropriate. Survival was analyzed using the Kaplan-Meier method. *P* < 0.05 was used to indicate statistical significance. Analyses were performed using PRISM 8.0.1 (GraphPad Software, San Diego, CA).

## Results

### SIRT3/5 Deficiency Has No Dramatic Impact on Mouse Development and Macrophage Metabolism

**Figure 1:**
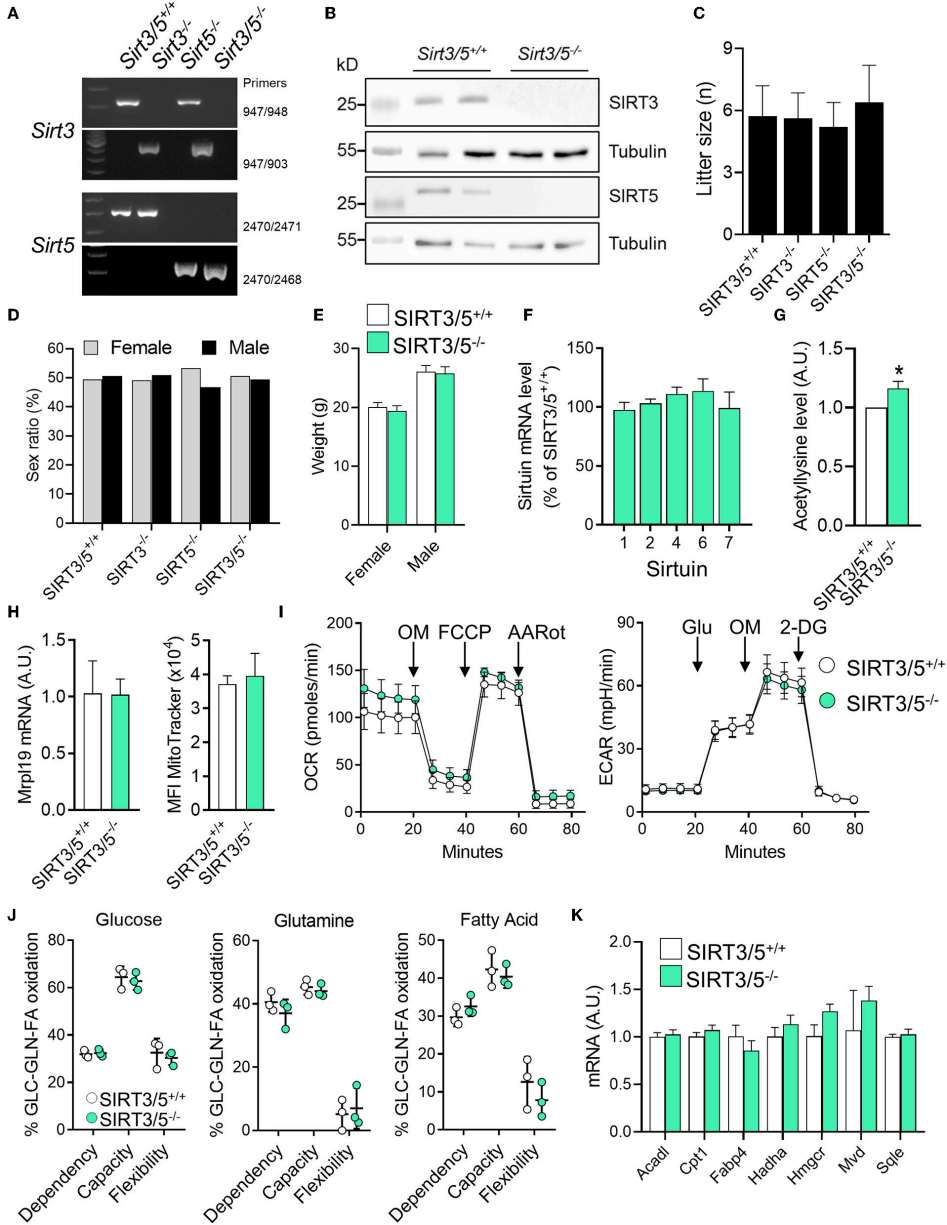
SIRT3/5 deficiency has no evident impact on mouse development and macrophage metabolism. **(A)** Genotyping of SIRT3/5^+/+^, SIRT3^−/−^, SIRT5^−/−^, and SIRT3/5^−/−^ mice by PCR. Genomic DNA was amplified by PCR using the primer pairs indicated on the right and reaction mixtures were electrophoresed through agarose gels. **(B)** SIRT3, SIRT5, and tubulin expression in the liver of SIRT3/5^+/+^ and SIRT3/5^−/−^ mice measured by Western blotting (Full blots are presented in Supplementary Figure S1). **(C,D)** Size **(C)** and sex ratio (in percentage, **D**) of litters from SIRT3/5^+/+^, SIRT3^−/−^, SIRT5^−/−^, and SIRT3/5^−/−^ mouse lines. **(E)** Weight of adult SIRT3/5^+/+^ and SIRT3/5^−/−^ female and male mice. **(F)** Sirtuin mRNA expression levels in SIRT3/5^−/−^ BMDMs, expressed relative to the mRNA levels in SIRT3/5^+/+^ BMDMs set at 100%. Data are means ± SD from one experiment performed with three mice analyzed in triplicate. **(G)** Acetyllysine levels in total protein extracts from SIRT3/5^+/+^ and SIRT3/5^−/−^ BMDMs were measured by Western blotting and imaging. Values were normalized to those obtained using SIRT3/5^+/+^ BMDMs set at 1. Data are means ± SD from one experiment performed with three mice. *P* = 0.009. **(H)** Mrpl19 mRNA expression levels assessed by RT-PCR and median fluorescence intensity (MFI) of MitoTracker measured by flow cytometry in SIRT3/5^+/+^ and SIRT3/5^−/−^ BMDMs. Data are means ± SD from four mice aged 10–12 weeks. **(I)** Oxygen consumption rate (OCR) and extracellular acidification rate (ECAR) were measured using the Seahorse technology. OM, oligomycin; FCCP, carbonyl cyanide 4-(trifluoromethoxy) phenylhydrazone; AARot, rotenone+Antimycin A; Glu, glucose; 2-DG, 2-deoxyglucose. Data are means ± SD from four mice aged 10–12 weeks analyzed in quadruplicate. **(J)** Mitochondrial fuel usage by SIRT3/5^+/+^ and SIRT3/5^−/−^ BMDMs measured using the Seahorse technology. **(K)** Acadl, Cpt1, Fabp4, Hadha, Hmgcr, Mvd, and Sqle mRNA expression levels were quantified by RT-PCR. Gene expression levels were normalized to actin levels. A.U., arbitrary unit. Data are means ± SD from three mice aged 10–12 weeks analyzed in triplicate.

We generated a SIRT3/5 double knockout mouse line (SIRT3/5^−/−^, see Materials and Methods) to study the interaction between SIRT3 and SIRT5. Genomic-DNA based PCR genotyping (Figure 1A) and Western blotting analyses (Figure 1B) confirmed the truncation of the *Sirt3* and *Sirt5* genes and the absence of SIRT3 and SIRT5 protein expression in SIRT3/5^−/−^ mice. Fecundity and development were normal. The size (Figure 1C) and the female/male sex ratio (Figure 1D) of the litters as well as the weight of adult female and male mice (Figure 1E) were like those of SIRT3/5^+/+^, SIRT3^−/−^, and SIRT5^−/−^ mouse lines. Autopsy did not reveal gross abnormalities in SIRT3/5^−/−^ mice.

The dual deletion of SIRT3 and SIRT5 was not compensated by an increased expression of mRNA encoding for SIRT1, SIRT2, SIRT4, SIRT6, and SIRT7 in bone marrow derived macrophages (BMDMs) (Figure 1F). There was around 20% increase of total protein acetylation in SIRT3/5^−/−^ BMDMs (Figure 1G). SIRT3/5 deficiency did not alter the mitochondrial mass, evaluated by measuring mitochondrial ribosomal protein L19 (Mrpl19) mRNA levels and the fluorescence intensity of the mitochondrial dye MitoTracker (Figure 1H). The oxygen consumption rate (OCR), which reflects mitochondrial respiration, was weakly increased in SIRT3/5^−/−^ BMDMs (Figure 1I). The extracellular acidification rate (ECAR), a readout of the glycolytic activity, was not affected in resting and LPS stimulated SIRT3/5^−/−^ BMDMs (Figure 1I and Supplementary Figure S3A). OCR and ECAR were similarly affected in resting SIRT3^−/−^ BMDMs (Supplementary Figure S3B) and SIRT5^−/−^ BMDMs (38). The dependency, capacity, and flexibility of BMDMs to oxidize the mitochondrial fuels glucose, glutamine and fatty acids were identical for SIRT3/5^+/+^ and SIRT3/5^−/−^ BMDMs (Figure 1J). SIRT3/5^+/+^ and SIRT3/5^−/−^ BMDMs expressed similar levels of a number of genes encoding for molecules involved in the fatty acid metabolism, i.e., Acadl (acyl-CoA dehydrogenase long chain), Cpt1 (carnitine palmitoyltransferase 1), Fabp4 (fatty acid binding protein 4), Hadha (hydroxyacyl-CoA dehydrogenase trifunctional multienzyme complex subunit alpha), Hmgcr (3-hydroxy-3-methylglutaryl-CoA reductase), Mvd (mevalonate diphosphate decarboxylase) and Sqle (squalene epoxidase) (Figure 1K). Finally, the OCR of SIRT3/5^+/+^ and SIRT3/5^−/−^ BMDMs was not different before and after addition of etimoxir, an inhibitor of Cpt1 (88.0 vs. 85.5% inhibition in SIRT3/5^+/+^ vs. SIRT3/5^−/−^ BMDMs; *n* = 6; *P* = 0.3). Hence, SIRT3/5 deficiency had no strong impact on basic metabolic parameters of BMDMs.

### SIRT3/5 Deficient Mice Have Minor Abnormalities of Leukocyte Development

**Figure 2:**
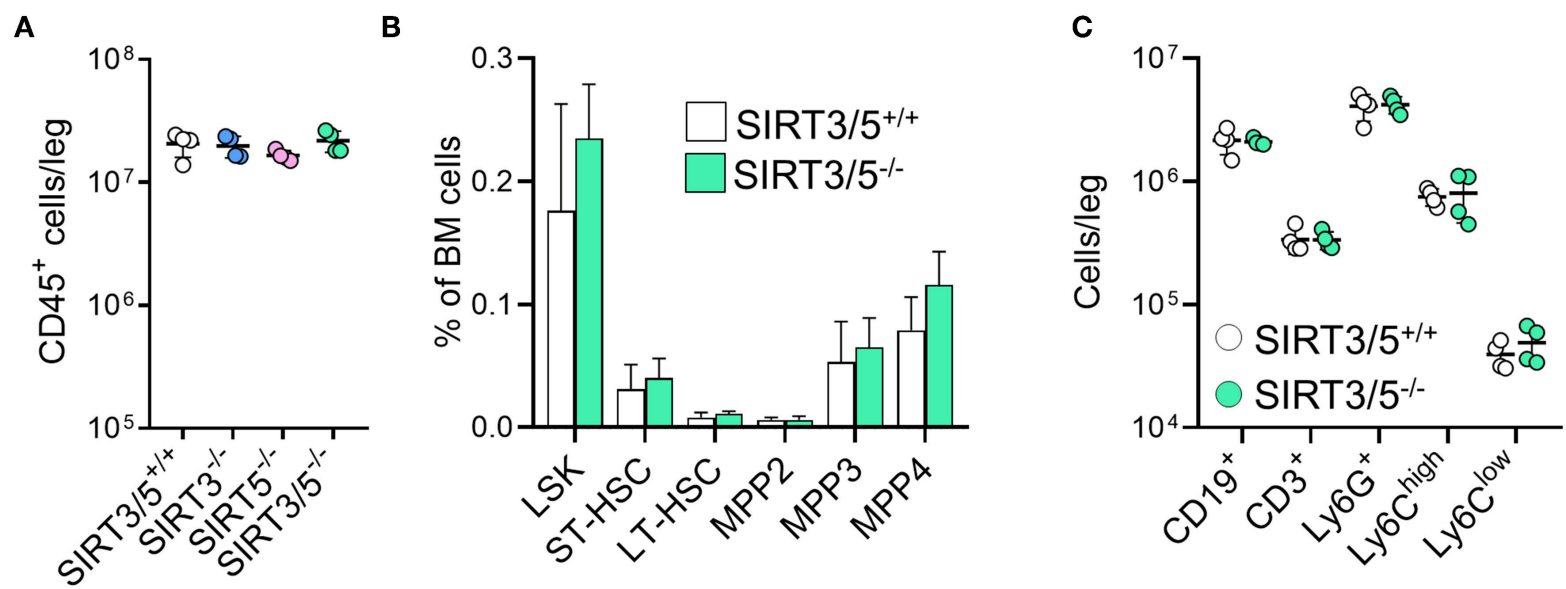
SIRT3/5 deficient mice have a normal number of hematopoietic stem cells and progenitors in the bone marrow. **(A)** Number of CD45^+^ bone marrow cells per leg of SIRT3/5^+/+^, SIRT3^−/−^, SIRT5^−/−^, and SIRT3/5^−/−^ mice aged 10–14 weeks. **(B)** Percentage of lineage negative, Sca1 positive, c-kit positive (LSK) cells, long-term (LT)-hematopoietic stem cells (HSC), short-term (ST)-HSC, multipotent progenitors (MPP), MPP2, MMP3, and MPP4 among BM cells. Data are means ± SD from eight mice. **(C)** Number of CD19^+^, CD3^+^, Ly6G^+^, Ly6C^high^, or Ly6C^low^ cells per leg of SIRT3/5^+/+^ and SIRT3/5^−/−^ mice.

The bone marrow is the main source of hematopoietic stem cells (HSC) and progenitors of immune cells during adulthood (54, 55). The number of CD45^+^ hematopoietic cells per leg (femur + tibia) was identical in SIRT3/5^+/+^, SIRT3^−/−^, SIRT5^−/−^, and SIRT3/5^−/−^ mice (Figure 2A), as well as the composition of the HSC pool which is made of lineage negative, Sca1 positive, c-kit positive (LSK) cells, long-term (LT)-HSC, short-term (ST)-HSC, and multipotent progenitors (MPP) (Figure 2B). Accordingly, the percentage and the absolute number of T cells, B cells, neutrophilic granulocytes and monocytes were similar in SIRT3/5^+/+^ and SIRT3/5^−/−^ mice, while some minor changes were observed in SIRT3^−/−^ and SIRT5^−/−^ mice (decreased CD3^+^ T cells and CD19^+^ B cells and increased monocytes) (Figure 2C and Table 1).

**Table 1:** Bone marrow leukocyte subsets.

	**SIRT3/5^**+/+**^ (*n* = 4)**	**SIRT3^**−/−**^ (*n* = 4)**	**SIRT5^**−/−**^ (*n* = 4)**	**SIRT3/5^**−/−**^ (*n* = 4)**
CD3^+^ T cells	2.8 ± 0.4	1.9 ± 0.5	1.6 ± 0.4	2.8 ± 0.5
CD19^+^ B cells	17.6 ± 1.0	14.8 ± 1.1	16.9 ± 2.8	17.3 ± 1.2
Ly6G^+^ Ly6C^−^ granulocytes	33.5 ± 4.2	39.4 ± 1.5	35.9 ± 4.3	34.6 ± 4.0
Ly6C^+^ Ly6G^−^ monocytes	6.8 ± 0.4	9.7 ± 1.7	7.0 ± 1.4	8.0 ± 1.9
Ly6C^high^ inflammatory/classical monocytes	93.8 ± 0.5	95.1 ± 0.3	93.2 ± 1.4	93.2 ± 0.8
Ly6C^low^ alternative/patrolling monocytes	6.2 ± 0.5	4.9 ± 0.3	6.9 ± 1.4	6.8 ± 0.8

*Data are means ± SD of four mice (aged 10–14 weeks) per group and expressed as the percentage of total cells (CD3^+^, CD19^+^, Ly6G^+^, Ly6C^+^) or the percentage of parental cells. Gray background: P <0.05 vs. SIRT3/5^+/+^*.

SIRT3/5^−/−^ mice, but not single knockout mice, showed a slight reduction of thymus cellularity when compared to SIRT3/5^+/+^ mice (SIRT3/5^+/+^ vs. SIRT3/5^−/−^ mice: 12.7 ± 1.8 vs. 8.3 ± 1.2 million cells; *P* = 0.03). However, the proportion of CD4/CD8 single positive, double positive and double negative (DN1-DN4) thymocytes was comparable in all mouse lines (Table 2). The size of the spleen (SIRT3/5^+/+^, SIRT3^−/−^, SIRT5^−/−^, and SIRT3/5^−/−^ mice: 6.1 ± 1.5, 5.3 ± 1.1, 7.3 ± 1.6, and 7.0 ± 1.8 × 10^7^ cells; *P* > 0.05 for all) and the proportion of total T cells, B cells, dendritic cells (DCs), neutrophilic granulocytes and monocytes were not affected in SIRT3/5^−/−^ mice (Table 3). Small, statistically significant, differences were noticed between SIRT3/5^+/+^ and SIRT3/5^−/−^ mice, i.e., reduced percentages of effector memory CD4^+^ T cells (23.1 ± 3.3 vs. 17.2 ± 2.3% of CD4^+^ T cells), CD4^−^CD8^−^ T cells (4.2 ± 0.9 vs. 3.0 ± 0.2% of CD3^+^ T cells) and conventional DC1 (cDC1, 35.4 ± 6.3 vs. 27.2 ± 3.0% of CD11c^+^ DCs) (Table 3). Like SIRT3/5^−/−^ mice, SIRT3^−/−^ mice showed a reduced percentage of cDC1, while SIRT5^−/−^ mice showed an increased percentage of Ly6C^low^ (alternative) monocytes at the expense of Ly6C^high^ (inflammatory) monocytes. Collectively, these results suggested that the dual deletion of SIRT3 and SIRT5 had a subtle impact on the development of immune cells.

**Table 2:** Thymic cell subsets.

	**SIRT3/5^**+/+**^ (*n* = 4)**	**SIRT3^**−/−**^ (*n* = 4)**	**SIRT5^**−/−**^ (*n* = 4)**	**SIRT3/5^**−/−**^ (*n* = 4)**
CD4^+^	13.0 ± 1.7	15.7 ± 0.7	16.1 ± 2.4	13.3 ± 1.6
CD8^+^	2.4 ± 0.4	3.4 ± 0.1	3.3 ± 0.7	2.9 ± 0.5
CD4^+^ CD8^+^	73.0 ± 1.6	67.4 ± 1.8	67.6 ± 3.2	71.9 ± 2.5
CD4^−^ CD8^−^	7.9 ± 0.9	8.9 ± 2.2	8.7 ± 1.3	8.3 ± 2.3
DN1: CD25^−^ CD44^+^	10.6 ± 1.8	12.2 ± 5.4	9.1 ± 1.9	10.7 ± 2.7
DN2: CD25^+^ CD44^+^	7.7 ± 1.1	8.9 ± 2.2	6.2 ± 1.2	6.4 ± 1.9
DN3: CD25^+^ CD44^−^	15.3 ± 2.1	16.8 ± 3.3	14.6 ± 2.2	16.0 ± 2.4
DN4: CD25^−^ CD44^−^	66.4 ± 4.8	62.1 ± 10.9	70.1 ± 4.5	66.8 ± 6.7

*Data are means ± SD of four mice (aged 7–8 weeks) per group and expressed as the percentage of total thymocytes (CD4^+^, CD8^+^, CD4^+^ CD8^+^, CD4^−^ CD8^−^) or the percentage of CD4^−^ CD8^−^ cells. No statistically significant differences in subsets' percentages were detected*.

**Table 3:** Splenic cell subsets.

	**SIRT3/5^**+/+**^ (*n* = 4)**	**SIRT3^**−/−**^ (*n* = 4)**	**SIRT5^**−/−**^ (*n* = 4)**	**SIRT3/5^**−/−**^ (*n* = 4)**
CD3^+^ T cells	21.5 ± 2.6	23.3 ± 3.9	25.0 ± 4.2	24.4 ± 1.5
CD4^+^	58.7 ± 4.8	56.0 ± 1.9	61.2 ± 2.0	60.9 ± 1.2
CD44^low^ CD62L^high^ naïve	43.0 ± 12.6	48.4 ± 10.3	53.7 ± 8.1	49.5 ± 7.4
CD44^high^ CD62L^low^ memory	23.1 ± 3.3	19.1 ± 2.7	20.2 ± 3.0	17.2 ± 2.3
CD8^+^	29.6 ± 5.6	34.0 ± 1.8	31.7 ± 0.7	32.2 ± 0.7
CD44^low^ CD62L^high^ naïve	70.4 ± 6.3	73.4 ± 4.7	71.4 ± 3.7	71.3 ± 5.1
CD44^high^ CD62L^low^ memory	3.6 ± 0.9	3.5 ± 0.7	4.8 ± 1.2	3.9 ± 1.0
CD4^−^ CD8^−^	4.2 ± 0.9	3.8 ± 0.5	3.3 ± 0.6	3.0 ± 0.2
CD4^+^ CD8^+^	7.6 ± 4.3	6.2 ± 2.1	3.8 ± 1.3	4.0 ± 0.8
B220^+^ B cells	56.3 ± 3.5	52.4 ± 3.9	58.6 ± 1.1	53.5 ± 2.1
IgD^−^ CD23^+^ mature	17.0 ± 5.3	18.0 ± 3.4	24.7 ± 2.9	16.7 ± 3.1
Non-IgD^+^/CD23^+^ immature	83.0 ± 5.3	82.0 ± 3.4	75.3 ± 2.9	83.3 ± 3.1
CD11c^+^ DCs	2.9 ± 0.4	3.0 ± 0.4	3.3 ± 0.1	2.8 ± 0.2
B220^+^ pDCs	19.1 ± 2.6	19.6 ± 4.8	21.0 ± 2.1	16.8 ± 1.6
B220^−^ cDCs	79.7 ± 2.9	78.9 ± 5.1	77.8 ± 2.2	82.0 ± 1.8
cDC1	35.4 ± 6.3	27.7 ± 5.4	33.6 ± 2.8	27.2 ± 3.0
cDC2	55.1 ± 6.4	63.0 ± 6.2	55.9 ± 3.4	63.4 ± 4.1
Ly6G^+^ Ly6C^−^ granulocytes	6.2 ± 5.5	5.5 ± 3.7	1.2 ± 0.3	5.9 ± 2.2
Ly6C^+^ Ly6G^−^ monocytes	3.7 ± 1.1	3.9 ± 0.7	3.1 ± 0.5	4.7 ± 0.8
Ly6C^high^	34.1 ± 14.7	25.5 ± 6.4	19.1 ± 3.5	35.9 ± 1.8
Ly6C^int^	26.0 ± 6.2	30.2 ± 4.0	33.2 ± 2.9	27.7 ± 1.7
Ly6C^low^	33.5 ± 9.5	37.1 ± 3.5	42.3 ± 2.6	31.1 ± 0.9

*Data are means ± SD of four mice (aged 10–14 weeks) per group and expressed as the percentage of total splenocytes (CD3^+^ T cells, B220^+^ B cells, CD11c^+^ DCs, Ly6G^+^ granulocytes, monocytes) or the percentage of parental cells. Gray background: P <0.05 vs. SIRT3/5^+/+^*.

### SIRT3/5 Deficiency Increases the Inflammatory Profile of Macrophages and the Killing Activity of Neutrophils

**Figure 3:**
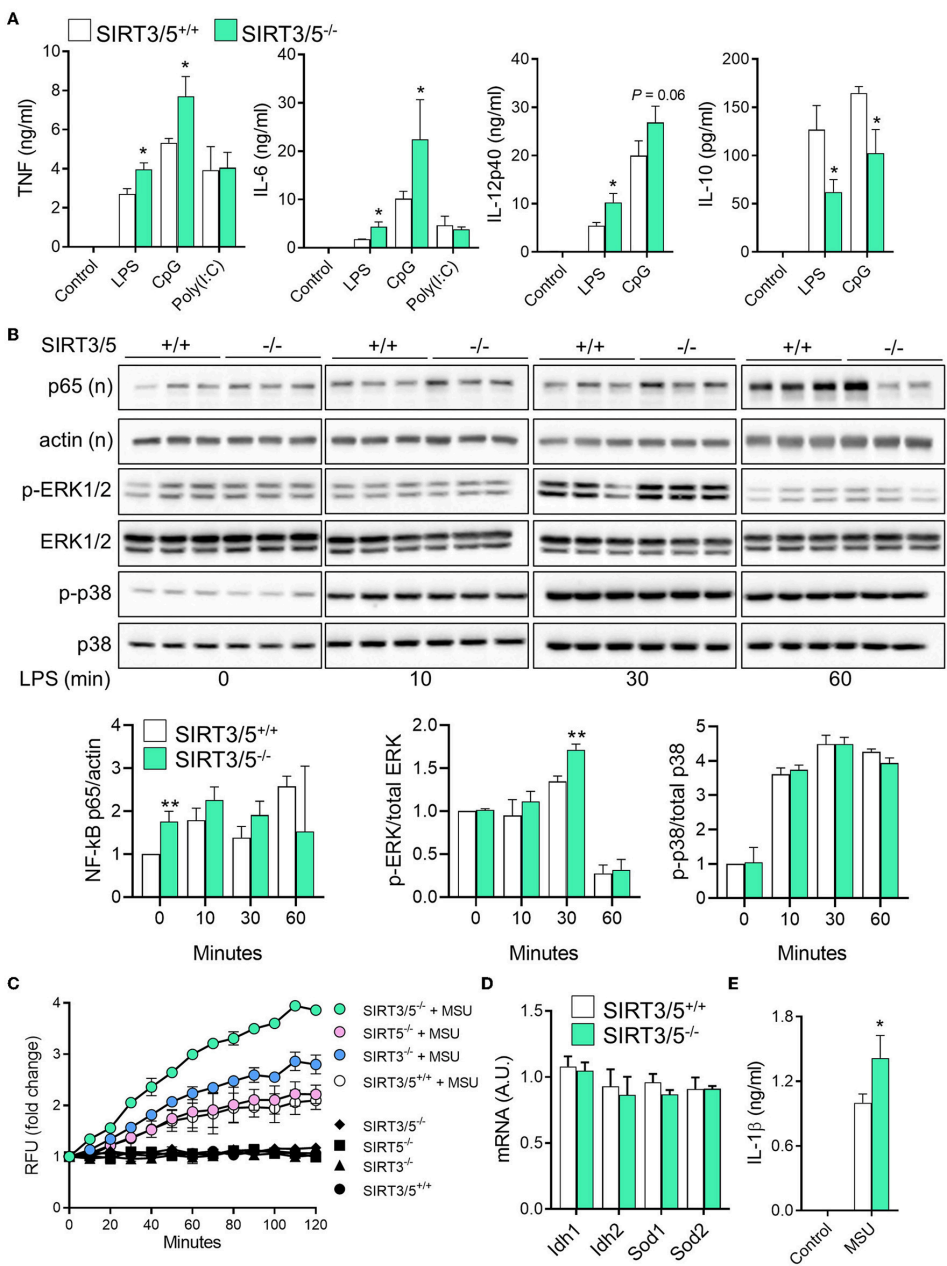
SIRT3/5 deficient macrophages display an enhanced proinflammatory profile. **(A)** SIRT3/5^+/+^ and SIRT3/5^−/−^ BMDMs were exposed for 24 h to LPS (10 ng/ml), CpG (1 μg/ml), and poly(I:C) (10 μg/ml). TNF, IL-6, IL-12p40, and IL-10 concentrations in cell culture supernatants were quantified by ELISA. Data are means ± SD from one experiment performed with four mice. **(B)** SIRT3/5^+/+^ and SIRT3/5^−/−^ BMDMs were exposed for 0, 10, 30, and 60 min to LPS (10 ng/ml). Nuclear (n) and total protein extracts were used to analyze NF-κB p65, and phosphorylated (p) and total ERK1/2 and p38. Signals were quantified by imaging and results (ratio p65/actin, p-ERK1/2/ERK1/2, and p-p38/p38) expressed relative to the results obtained in resting SIRT3/5^+/+^ BMDMs set at 1. Data are means ± SD from one experiment performed with three mice. Full blots are presented in Supplementary Figure S1. **(C–E)** BMDMs were primed with Pam_3_CSK_4_ (10 ng/ml) for 18 h and exposed (colored and white symbols) or not (black symbols) to MSU crystals for the indicated time **(C)** or 6 h **(E)**. mtROS were quantified using MitoSOX **(C)**, Idh1, Idh2, Sod1, and Sod2 mRNA levels by RT-PCR **(D)** and IL-1β by ELISA **(E)**. Gene expression levels were normalized to actin levels. A.U, arbitrary unit. Data are mean ± SD of four mice aged 10–12 weeks analyzed in triplicate **(C–E)**. ^*^P < 0.05; ^**^P < 0.01.

Macrophages and neutrophils are proficient at sensing microbial products and play key defense roles during infections. SIRT3 and SIRT5 single deficiency did not influence antimicrobial host defenses (37, 38). Therefore, we asked whether dual deficiency of SIRT3 and SIRT5 would reveal a phenotype unseen in single knockouts. SIRT3/5^+/+^ and SIRT3/5^−/−^ BMDMs were exposed to LPS, CpG, and poly(I:C), which are sensed through TLR4, TLR9, and TLR3, respectively. SIRT3/5^−/−^ BMDMs produced higher levels of TNF, IL-6, and IL-12p40 (as a trend for CpG-induced IL-12p40) and lower levels of IL-10 than SIRT3/5^+/+^ BMDMs in response to LPS and CpG, while SIRT3/5^+/+^ and SIRT3/5^−/−^ BMDMs produced similar levels of TNF and IL-6 in response to poly(I:C) (Figure 3A).

To address whether the increased inflammation driven by SIRT3/5-deficiency was linked to increased intracellular signaling, we quantified by Western blotting the nuclear translocation of NF-κB p65 and the phosphorylation of ERK1/2 and p38 MAPKs in BMDMs exposed for 0, 10, 30, and 60 min to LPS (Figure 3B). In SIRT3/5^−/−^ BMDMs, there was an increased NF-κB p65 nuclear content at baseline and, albeit not statistically significant, after 30 and 60 min of stimulation. The level of phospho-ERK1/2 was also increased after 30 min of exposure to LPS. Overall, SIRT3/5 deficiency increased inflammatory intracellular signaling pathways and inflammatory cytokine production by BMDMs.

ROS activate the NOD-like receptor pyrin domain-containing-3 (NLRP3) inflammasome that cleaves pro-IL-1β into mature IL-1β that is secreted. Considering that SIRT3 and SIRT5 activate enzymes playing a role in the detoxification process of ROS (i.e., IDH1, IDH2, SOD1, and SOD2) (17–21), we questioned whether SIRT3/5^−/−^ BMDMs produce increased levels of mitochondrial ROS (mtROS) and IL-1β. SIRT3/5^+/+^, SIRT3^−/−^, SIRT5^−/−^, and SIRT3/5^−/−^ BMDMs were exposed to monosodium urate (MSU) crystals, a commonly used activator of the NLRP3 inflammasome before measuring mtROS (Figure 3C). MSU crystals induced mtROS equally in SIRT3/5^+/+^ and SIRT5^−/−^ BMDMs, 1.5-fold more in SIRT3^−/−^ BMDMs and 2-fold more in SIRT3/5^−/−^ BMDMs. mRNA levels of Idh1, Idh2, Sod1, and Sod2 were similar in SIRT3/5^+/+^ and SIRT3/5^−/−^ BMDMs (Figure 3D), in agreement with the fact that sirtuins target the activity rather than the expression of IDH1, IDH2, SOD1, and SOD2. As expected from the above, SIRT3/5^−/−^ BMDMs secreted higher levels of IL-1β than SIRT3/5^+/+^ BMDMs (Figure 3E).

**Figure 4:**
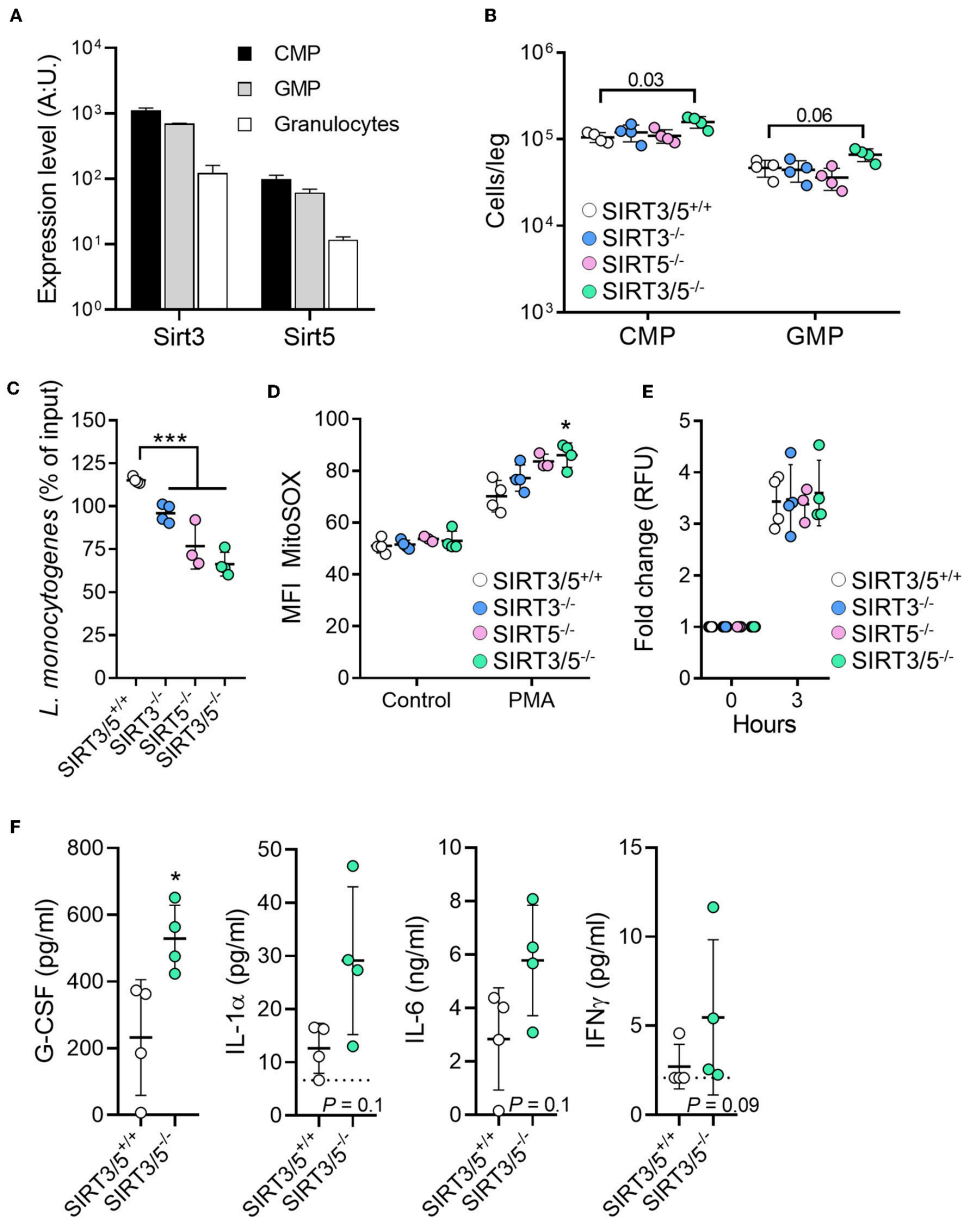
Increased killing of *Listeria monocytogenes* by SIRT3/5 deficient neutrophils. **(A)** Sirt3 and Sirt5 mRNA expression levels in common myeloid progenitors (CMP), granulocyte-monocyte progenitor (GMP), and granulocytes. Data were extracted from BioGPS (http://biogps.org). **(B)** Number of CMP (Lin^−^ c-kit^+^ CD34^+^ cells) and GMP (Lin^−^ c-kit^+^ CD135^+^ CD34^+^ cells) per leg of SIRT3/5^+/+^, SIRT3^−/−^, SIRT5^−/−^, and SIRT3/5^−/−^ mice aged 8–9 weeks. The gating strategies are presented in Supplementary Figure S2. **(C–E)** Killing of bacteria **(C)** and production of mtROS **(D)** and NETs **(E)** by neutrophils. Neutrophils were incubated for 1 h with *L. monocytogenes* (0.1 cfu/cell) **(C)**, for 50 min with PMA (100 nM) and MitoSOX **(D)** or for 3 h with PMA (100 nM) and SYTOX **(E)**. Bacteria were enumerated and results expressed relative to the initial inoculum set at 100% **(C)**. ^*^P < 0.05; ^***^P < 0.005. **(F)** G-CSF, IL-1α, IL-6, and IFNγ concentrations, determined by Luminex, in blood from SIRT3/5^+/+^ and SIRT3/5^−/−^ mice incubated with heat-killed *L. monocytogenes* for 24 h. The concentrations of CCL2, CCL3, CXCL1, CXCL5, CXCL10, IL-1β, IL-10, IL-12p40, IL-17A, IL-18 and TNF were similar for SIRT3/5^+/+^ and SIRT3/5^−/−^ blood.

The role of sirtuins in the development and functions of granulocytes is scarce and has not been reported for SIRT5 (56, 57). The mRNA expression levels of SIRT3 and SIRT5 decreased gradually from common myeloid progenitors (CMP) to granulocyte-monocyte progenitor (GMP; 1.6-fold) and from GMP to neutrophilic granulocytes (5.3–5.7-fold) (Figure 4A). Compared to SIRT3/5^+/+^ mice, SIRT3/5^−/−^ mice expressed in the bone marrow statistically significantly more CMP (*P* = 0.03) but not GMP (*P* = 0.06) (Figure 4B). Accordingly, SIRT3/5^−/−^ mice expressed normal numbers of neutrophilic granulocytes in the bone marrow and spleen (Figure 2C and Table 3). We then addressed whether SIRT3/5 deficiency affected neutrophil functions. We setup a killing assay in which neutrophils were incubated for 1 h with *Listeria monocytogenes* before quantifying bacteria. As shown in Figure 4C, 115, 96, 77, and 66% of the starting inoculum were recovered from assays using SIRT3/5^+/+^, SIRT3^−/−^, SIRT5^−/−^, and SIRT3/5^−/−^ neutrophils, respectively. Hence, the dual deletion of SIRT3 and SIRT5 promoted the killing of *L. monocytogenes* by neutrophils. This prompted us to analyze two main mechanisms through which neutrophils kill bacteria, i.e., the production of ROS and the release of neutrophil extracellular traps (NETs). SIRT3/5^−/−^ neutrophils, and to a lesser extent SIRT3^−/−^ and SIRT5^−/−^ neutrophils, produced increased levels of ROS when compared to SIRT3/5^+/+^ neutrophils (Figure 4D). In contrast, SIRT3/5^+/+^, SIRT3^−/−^, SIRT5^−/−^, and SIRT3/5^−/−^ neutrophils produced similar amounts of NETs (Figure 4E). Thus, the proficient killing of *L. monocytogenes* by SIRT3/5^−/−^ neutrophils was more likely related to an increased generation of ROS than NETs. Lastly, we measured by Luminex the cytokines released by whole blood exposed to heat-killed *L. monocytogenes*. Fifteen of 17 mediators were produced at measurable levels: CCL2, CCL3, CXCL1, CXCL5, CXCL10, G-CSF, IFNγ, IL-1α, IL-1β, IL-6, IL-10, IL-12p40, IL-17A, IL-18, and TNF. Going well along with an increased inflammatory response of BMDMs (Figure 3), SIRT3/5^−/−^ whole blood produced more G-CSF and showed a trend toward producing more IL-1α, IL-6, and IFNγ than SIRT3/5^+/+^ whole blood (Figure 4F). The production of other cytokines was not affected.

### SIRT3/5 Deficiency Provides a Modest Protection to Listeriosis

**Figure 5:**
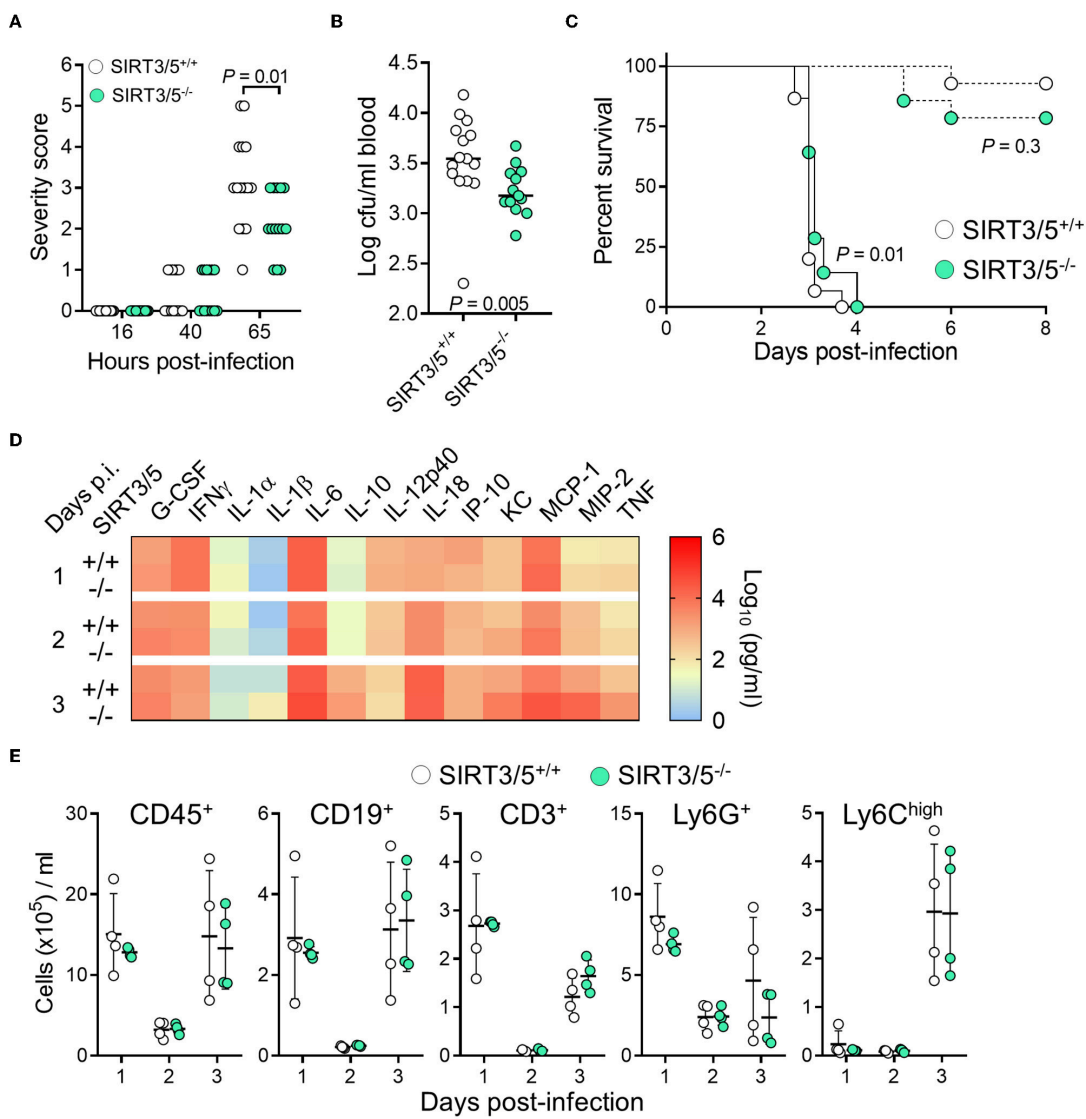
SIRT3/5 deficiency confers some protection to listeriosis. **(A–E)** SIRT3/5^+/+^ and SIRT3/5^−/−^ mice (*n* = 14–15 per group, aged 8–12 weeks) were injected intravenously with 7.3 × 10^3^ cfu (**C**, dashed lines) or 0.9–1.5 × 10^5^ cfu *L. monocytogenes* (**A–E**, plain lines in **C**). **(A)** Severity scores were recorded. **(B)** *L. monocytogenes* in blood collected 2 days post-infection. **(C)** Survival of mice. **(D)** Cytokines, quantified by Luminex, in blood collected 1, 2, and 3 days post-infection (p.i.). The horizontal bar represents the median. Data are mean ± SD of four mice per group. *P* = 0.001 for TNF at day 1 p.i. 0.004 and 0.03 for TNF and IL-1β at day 2 p.i. and 0.05 for KC at day 3 p.i. ENA-78, IL-3, IL-17A, and MIP-1α were not detected. **(E)** CD45^+^, CD19^+^, CD3^+^, Ly6G^+^ and Ly6C^high^ leukocytes in blood collected 1, 2, and 3 days post-infection. Each dot represents a mouse, and line and bars mean ± SD.

Myeloid cells play a crucial role in protecting from *L. monocytogenes* infection (58, 59). Considering that SIRT3/5 deficiency increased *L. monocytogenes* killing by neutrophils (Figure 4C) and cytokine production by macrophages and to some extent by whole blood (Figures 3A, 4F), we tested the relevance of these observations *in vivo* using a model of listeriosis. Mice challenged intravenously with a high inoculum of *L. monocytogenes* (0.9–1.5 × 10^5^ cfu) exhibited signs of disease 36–40 h post-infection and were severely sick after 65 h as shown by elevated severity scores in most animals. SIRT3/5^−/−^ mice were not as ill as SIRT3/5^+/+^ mice (*P* = 0.01; Figure 5A) and had 2.3-fold less *L. monocytogenes* in blood collected 48 h after infection (SIRT3/5^+/+^ vs. SIRT3/5^−/−^: 3.5 ± 0.9 × 10^3^ cfu/ml vs. 1.5 ± 0.3 × 10^3^ cfu/ml; median ± SEM; *P* = 0.005) (Figure 5B). In line with these observations, SIRT3/5^−/−^ mice had a modest, statistically significant, delayed mortality rate compared to SIRT3/5^+/+^ mice (median survival of SIRT3/5^+/+^ vs. SIRT3/5^−/−^: 3.0 vs. 3.12 days; *P* = 0.01) (Figure 5C). Going well along with an improved response to infection, SIRT3/5^−/−^ mice had significantly higher blood concentrations of TNF at day 1 (*P* = 0.001), TNF and IL-1β at day 2 (*P* = 0.004 and 0.03) and KC/CXCL1 at day 3 (*P* = 0.05). Albeit not statistically significant, the levels of G-CSF, IL-1α, MCP-1/CCL2, MIP-2/CXCL2 at day 1, G-CSF, KC, MCP-1, and MIP-2 at day 2, and IL-1α, IL-1β, IL-6, IL-10, MCP-1, MIP-2, and TNF at day 3 post-infection were 1.5–4.4-fold higher in SIRT3/5^−/−^ than in SIRT3/5^+/+^ mice (Figure 5D). As expected, 2 days after the onset of infection, listeriosis induced a dramatic drop of circulating leukocytes including B cells, T cells, neutrophils and Ly6C^high^ inflammatory monocytes that rebounded at day 3 (Figure 5E). No differences were observed between SIRT3/5^+/+^ and SIRT3/5^−/−^ mice, suggesting that the reactivity rather than the number of blood leukocytes conferred some protection to SIRT3/5^−/−^ mice during listeriosis. Interestingly, there was no statistically significant difference of mortality when SIRT3/5^−/−^ and SIRT3/5^+/+^ mice were challenged with a low inoculum (7.3 × 10^3^ cfu) of *L. monocytogenes* responsible for <25% death (Figure 5C). Finally, we tested the mouse lines in a model of endotoxemia induced by an intraperitoneal challenge with 10 mg/kg LPS. Surprisingly, there was no statistically significant difference between the SIRT3/5^+/+^ and SIRT3/5^−/−^ groups (*n* = 21–22 mice per group; *P* = 0.1).

## Discussion

This is the first report about the impact of the dual deficiency of SIRT3 and SIRT5 on immune cell development and antimicrobial host defenses. Double knockout mice developed normally and showed subtle, minor alterations of immune cell subpopulations and host responses to infection. Together with the fact that SIRT3^−/−^ and SIRT5^−/−^ mice are susceptible to bacterial sepsis like wild-type mice (37–40), these observations strengthen the development of pharmacological modulators of the activity of mitochondrial sirtuins for clinical purposes.

Notwithstanding that SIRT3 and SIRT5 orchestrate metabolism and oxidative stress responses, SIRT3 and SIRT5 whole body knockout mice have no macroscopic abnormalities (41, 42). The SIRT3/5^−/−^ mouse line we generated here developed normally. No problem of fertility, sex distribution and growth were noticed. Surprisingly, the metabolism of SIRT3/5^−/−^ BMDMs was similar to that of SIRT3/5^+/+^ BMDMs. Yet, the impact of SIRT3 and SIRT5 on metabolism was mainly demonstrated in cells or tissues such as the liver and the heart that are rich in mitochondrial sirtuins when compared to macrophages (38, 41, 60). Another SIRT3/5^−/−^ mouse line has been recently generated. In line with our observations, no developmental defects were reported. Moreover, these SIRT3/5^−/−^ mice were susceptible to streptozotocin-induced hyperglycemia like controls, while showing only a modest inner retinal dysfunction (61, 62).

Studies on the role of sirtuins in hematopoiesis and immune cell development are scarce. SIRT3/5^−/−^ mice had a normal pool of HSCs and MPPs and a slightly increased number of CMP (and GMP as a trend) in their bone marrow. The primary and secondary immune organs of SIRT3/5^−/−^ mice were largely unaffected, according to absolute numbers and proportions of immune cell subpopulations. There was only a slight reduction of thymus size, which did not impact the proportion of thymocyte subpopulations. This reminds the phenotype of SDHD-ESR mice with deletion of the *succinate dehydrogenase, subunit D* gene encoding for one of the subunits of the mitochondrial complex II (63). SDHD mice have a thymic atrophy without perturbation of thymocyte development. Overall, deficiencies in SIRT3, SIRT5, and SIRT3/5 do not seem to have a dramatic impact on immune cell development and/or functions [(37, 38) and present study]. Nonetheless, a role for these enzymes might come to light under stress or stimulatory conditions, or in aged mice. For example, SIRT1 shaped the T helper (Th) and T regulatory (Treg) responses of naïve T cells (64–68). Furthermore, in SIRT1^−/−^ mice, the percentages of CD4^+^, CD8^+^, and CD4^+^CD8^+^ T cell subpopulations were normal, but thymocytes were at increased sensitivity to ionizing radiation induced DNA damaging (69). Finally, SIRT3^−/−^ mice of 18–24 months had a lower frequency of bone marrow hematopoietic progenitors than mice of 12 weeks (70).

SIRT3/5^−/−^ macrophages exposed to TLR agonists produced more inflammatory cytokines and less IL-10 than SIRT3/5^+/+^ macrophages, contrary to SIRT3^−/−^ and SIRT5^−/−^ macrophages that behaved like wild-type cells (37, 38). Accordingly, NF-κB and MAPK signaling pathways were increased in resting and/or LPS-stimulated SIRT3/5^−/−^ macrophages. These data somehow support the possibility that SIRT3 and SIRT5 compensate each other in single knockout animals. Sirtuins are generally considered to drive anti-inflammatory responses. However, as nicely reviewed recently for SIRT1 (68), sirtuins may promote proinflammatory and anti-inflammatory effects depending on the context and whether myeloid or lymphoid cells are considered. For example, SIRT5 deficiency was associated with both increased and decreased innate inflammatory response *in vivo* (33, 71). More generally, contrasting observations have been reported for most sirtuins (SIRT1-3, SIRT5-6). Methodological differences may explain these differences when studying monocytes/macrophages (37, 38, 46): the origin/fate of the cells (BMDMs vs. peritoneum macrophages vs. established macrophage cell lines, growth factors used for differentiation and maturation state of macrophages), strategies to delete or overexpress sirtuins or to modulate sirtuin activity (siRNA, shRNA, expression vectors, full or cell-specific knockouts, pharmacological activators, and inhibitors), readouts, and subtle variations in NAD^+^ concentrations and circadian rhythm known to affect sirtuin activity or expression.

The expression levels of SIRT3 and SIRT5 decreased gradually from CMP to GMP and from GMP to granulocytes, which mirror the decline of mitochondrial mass and mitochondrial DNA during hematopoietic differentiation (72). Granulopoiesis relies on the expression of CCAAT/enhancer binding protein (C/EBP). The expression of SIRT1, which deacetylates C/EBPε and represses neutrophil terminal differentiation (73), declines during granulopoiesis (727 ± 13, 643 ± 20, and 307 ± 61 mRNA arbitrary units in CMP, GMP, and granulocytes, respectively). Thus, the downregulation of sirtuins seems to be a general feature associated with neutrophil development. The fact that neutrophil counts were normal and not increased in SIRT3/5^−/−^ mice suggests either the implementation of compensatory mechanisms, possibly through SIRT1, or that SIRT3 and SIRT5 have a modest influence on granulopoiesis.

Neutrophils produce cytotoxic compounds that target pathogenic bacteria and fungi but are harmful for host tissues (74). Contrary to SIRT3/5^+/+^ neutrophils, and better than SIRT3^−/−^ and SIRT5^−/−^ neutrophils, SIRT3/5^−/−^ neutrophils killed *L. monocytogenes*, which was associated with an augmented production of ROS but not of NETs. In comparison, neutrophils deficient in SIRT3 had a mild increase of intracellular ROS but performed either normal or increased NETosis (56, 57). SIRT1 deficiency did not impact on neutrophil functions, and the role of the remaining sirtuins has not been reported. Whereas, SIRT3^−/−^ mice had increased neutrophil infiltration in lungs during sterile injury (36, 75) and mycobacterial infection impairing the survival of mice (76), SIRT5^−/−^ had reduced inflammation and ischemia/reperfusion brain injury (77). Finally, SIRT3^−/−^ and SIRT5^−/−^ mice behaved like wild-type mice in models of sepsis requiring neutrophils to fight the infectious agents (37–40). Interestingly, SIRT3/5^−/−^ mice resisted better than SIRT3/5^+/+^ mice to acute listeriosis, showing decreased signs of morbidity, reduced blood bacterial loads and significant albeit modest delayed mortality. SIRT3/5^−/−^ mice expressed higher concentrations of cytokines but normal counts of leukocytes in blood, suggesting that the reactivity rather than the number of leukocytes protected SIRT3/5^−/−^ mice from listeriosis. Interestingly, SIRT3/5^−/−^ mice were not more resistant to mild listeriosis than their wild type counterparts, and behaved like wild type mice in a model of endotoxemia. These observations support the assumption that drugs targeting SIRT3 and SIRT5 should not have a deletary impact on host defenses, which would contrast with drugs targeting classical HDACs that strongly impaired innate immune defenses against infections in preclinical models and clinical settings (78–84).

Overall, the double deficiency in SIRT3 and SIRT5 had rather modest and subtle impacts on immune cell development and anti-microbial host defenses. It might be that SIRT4, the remaining mitochondrial sirtuin in SIRT3/5^−/−^ mice, compensated for SIRT3 and SIRT5 absence. Unfortunately, whether SIRT4 affects immune responses has not been reported. Considering the link between sirtuins, metabolism and age-associated pathologies, it is possible that phenotypes will emerge in aged mice or in mice submitted to metabolic stress. SIRT3/5^−/−^ mice should be tested in other preclinical models of sepsis. Nonetheless, putting together the data from the present study together with the fact that single deficiencies in SIRT3 and SIRT5 had no impact in a large panel of experimental sepsis (37–40), one may foresee that therapies directed against mitochondrial sirtuins or concomitant targeting of SIRT3 and SIRT5 activity should have no deep impact on antibacterial host defenses.

## Data Availability Statement

All datasets generated for this study are included in the manuscript/Supplementary Files.

## Ethics Statement

The animal study was reviewed and approved by Service des Affaires Vétérinaires, Direction Générale de l'Agriculture, de la Viticulture et des Affaires Vétérinaires (DGAV), état de Vaud (Epalinges, Switzerland; authorizations 876.9 and 877.9).

## Author Contributions

TH and EC performed *in vitro* experiments. TH, EC, and DLR performed *in vivo* experiments. TR and TH conceived the project, designed the experiments and wrote the paper. All the authors revised the paper.

### Conflict of Interest

The authors declare that the research was conducted in the absence of any commercial or financial relationships that could be construed as a potential conflict of interest.

## Supplementary Material

Supplementary Figure S1 Full blots used to extract the panels shown in Figures 1B, 3B.

Supplementary Figure S2 Gating strategies for flow cytometry analysis.CD117 encodes for c-kit.

Supplementary Figure S3 **(A)** Extracellular acidification rate (ECAR) by SIRT3/5^+/+^ and SIRT3/5^−/−^ BMDMs exposed to LPS (10 ng/ml) measured using the Seahorse technology. **(B)** Oxygen consumption rate (OCR) and ECAR by SIRT3/5^+/+^ and SIRT3^−/−^ BMDMs measured using the Seahorse technology. Data are means ± SD from three mice aged 10–12 weeks analyzed in quadruplicate.
