# Supplementary figures and images for: Impact of the Dual Deletion of the Mitochondrial Sirtuins SIRT3 and SIRT5 on Anti-microbial Host Defenses
Source: Front Immunol. 2019 Oct 1;10:2341. doi: 10.3389/fimmu.2019.02341 (PMC6781768; doi:10.3389/fimmu.2019.02341)

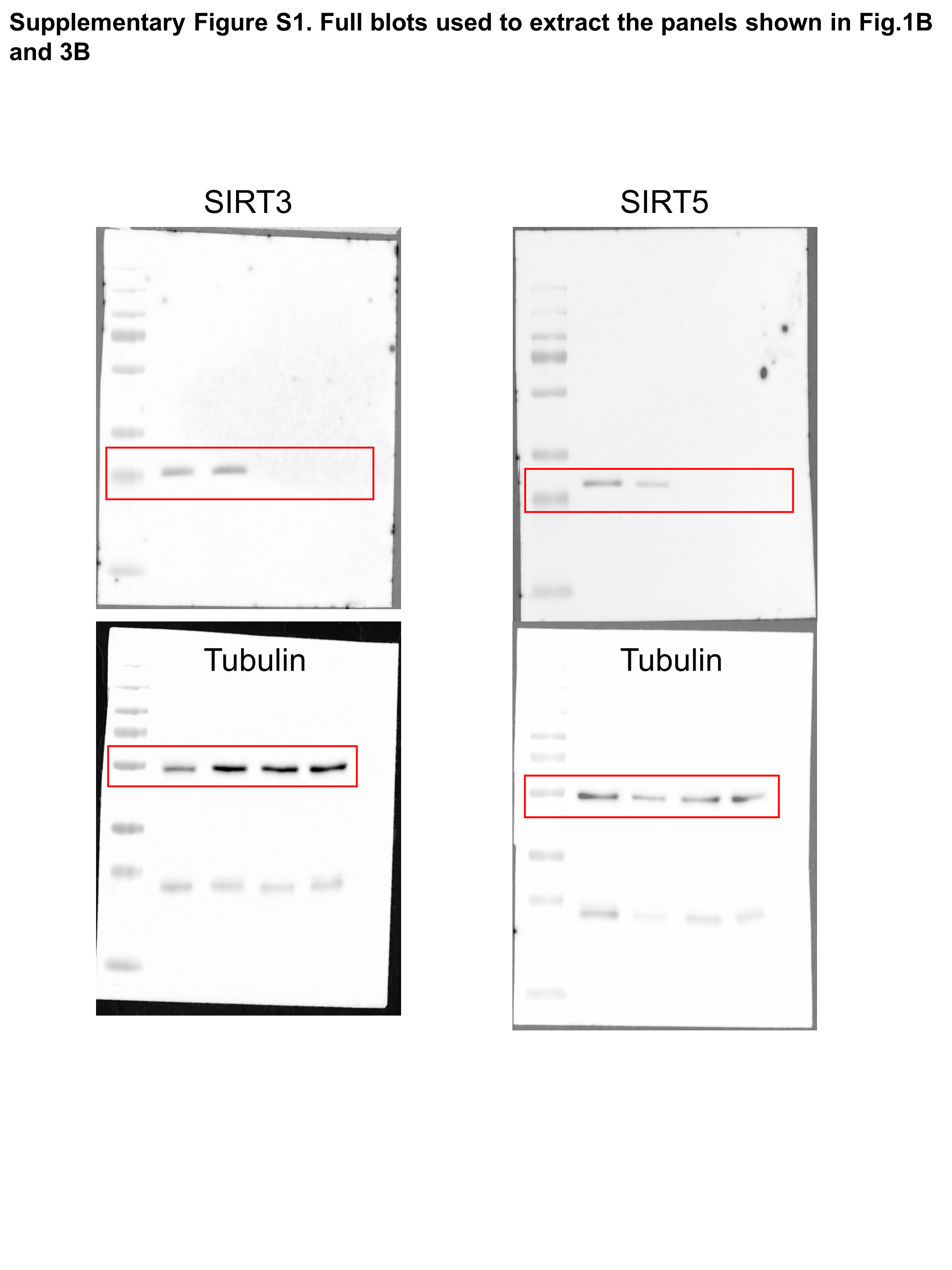

Supplement: Supplementary Figure S1 — Full blots used to extract the panels shown in Figures 1B, 3B. [file Presentation_1.zip › Image 1.1.TIF]

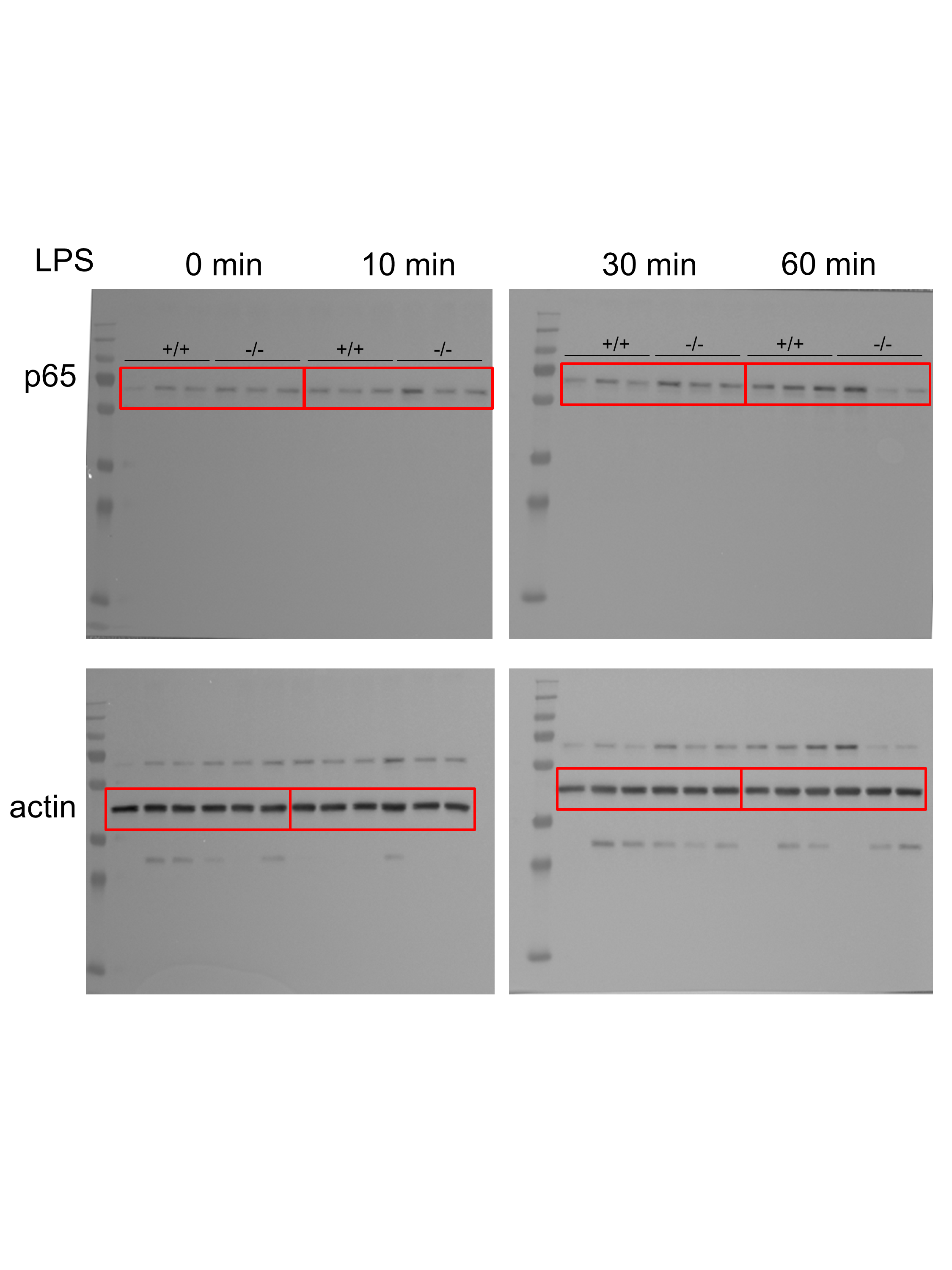

Supplement: Supplementary Figure S1 — Full blots used to extract the panels shown in Figures 1B, 3B. [file Presentation_1.zip › Image 1.2.TIF]

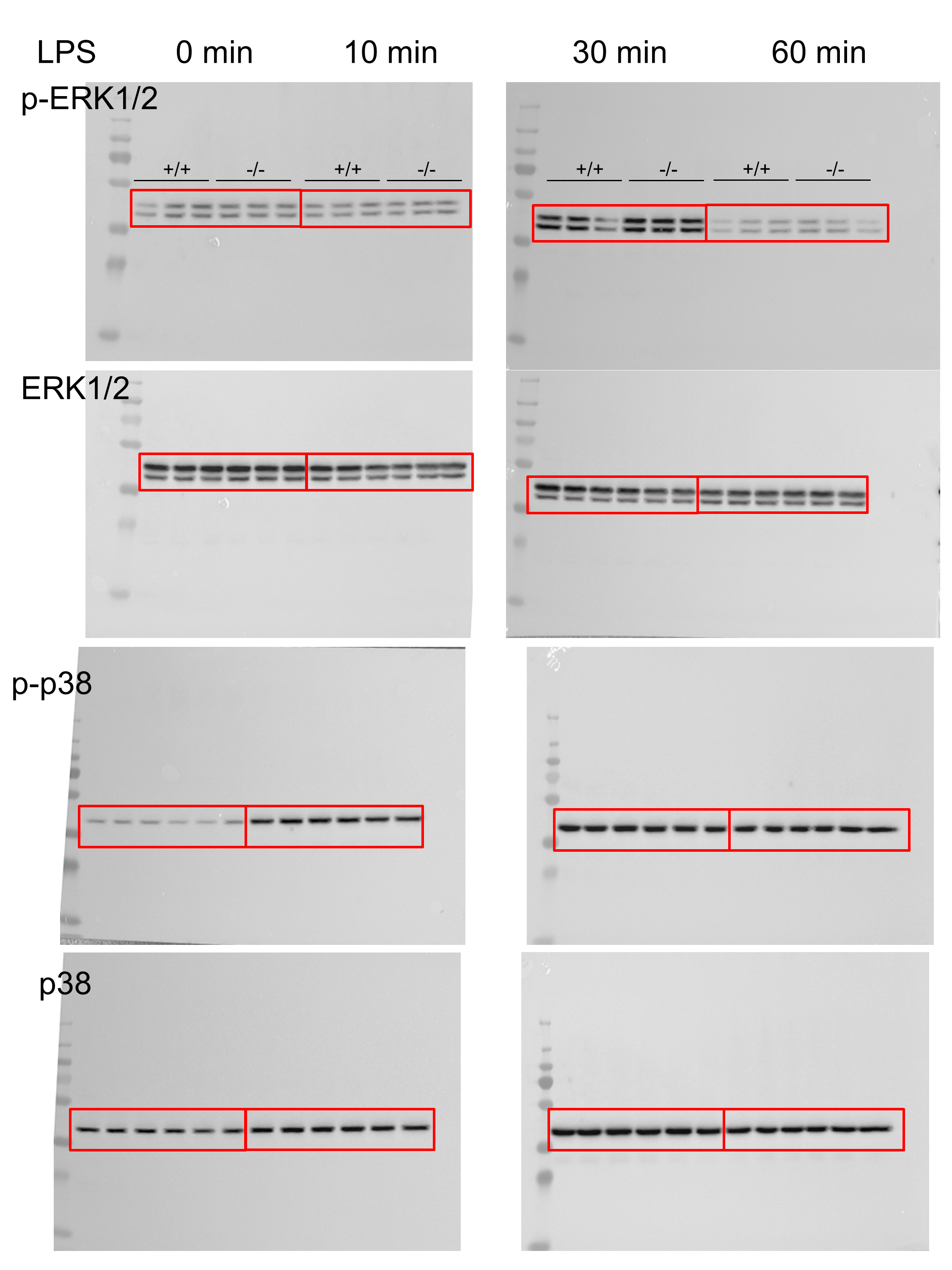

Supplement: Supplementary Figure S1 — Full blots used to extract the panels shown in Figures 1B, 3B. [file Presentation_1.zip › Image 1.3.TIF]

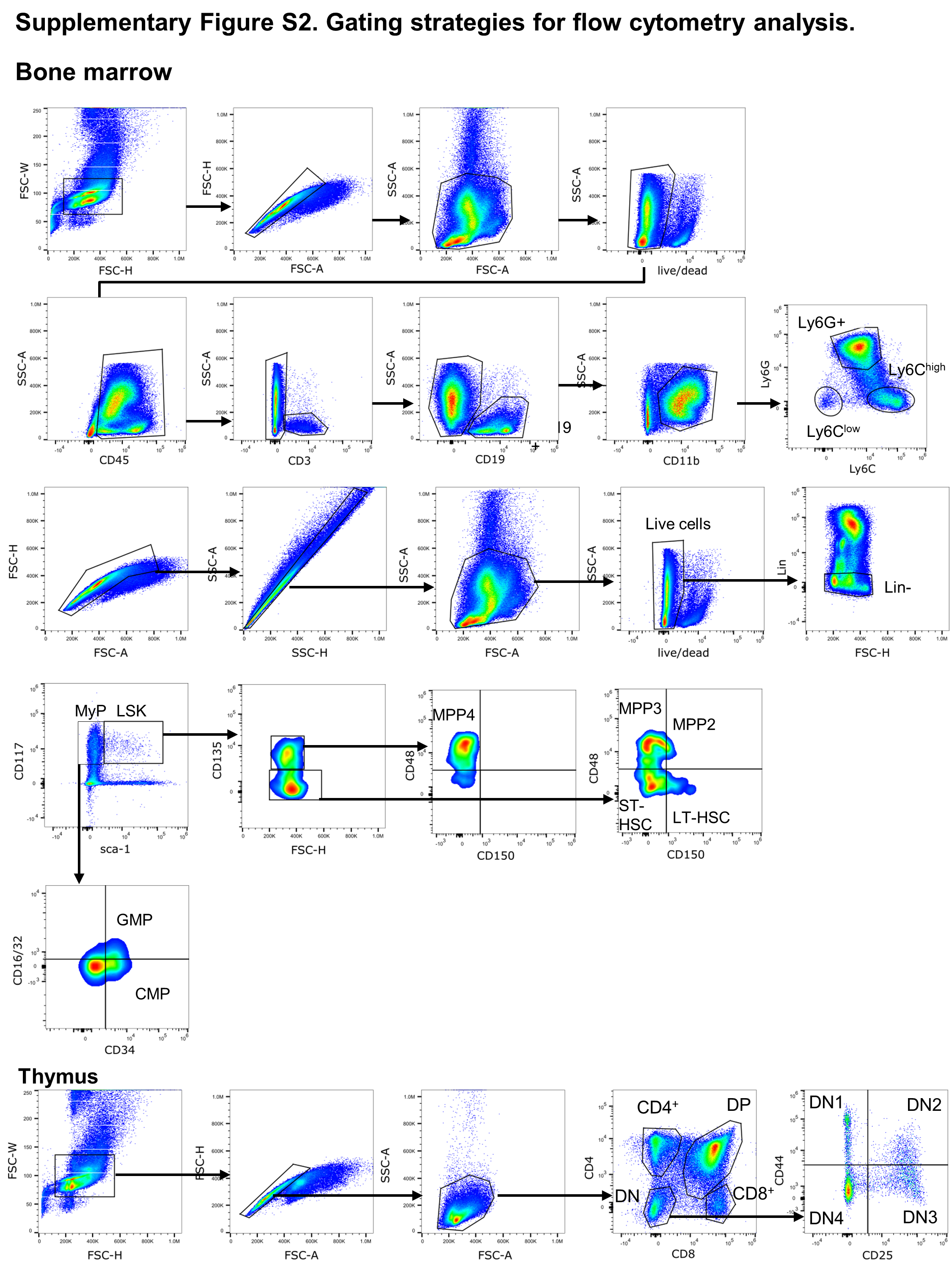

Supplement: Supplementary Figure S1 — Full blots used to extract the panels shown in Figures 1B, 3B. [file Presentation_1.zip › Image 2.1.TIF]

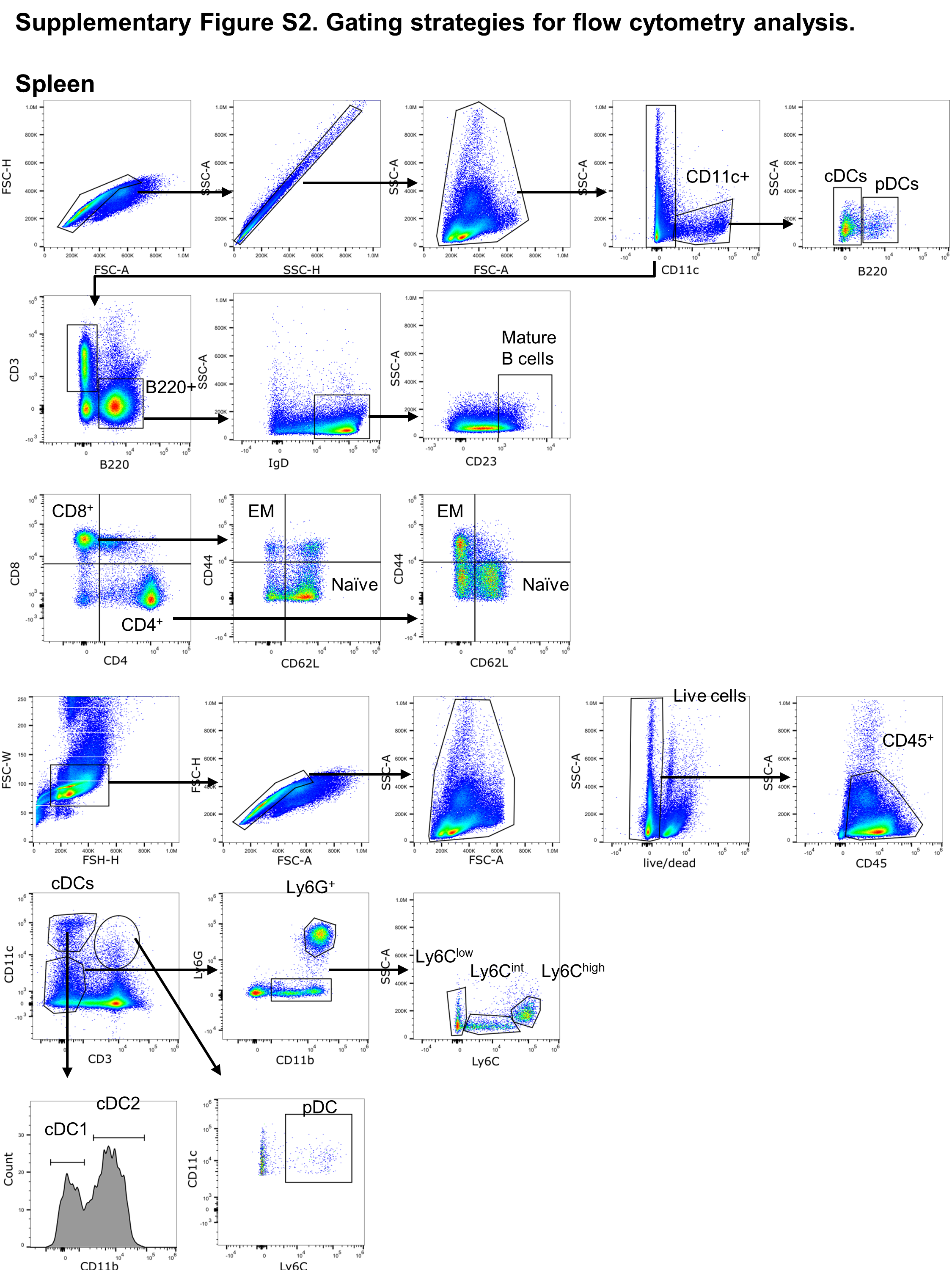

Supplement: Supplementary Figure S1 — Full blots used to extract the panels shown in Figures 1B, 3B. [file Presentation_1.zip › Image 2.2.TIF]

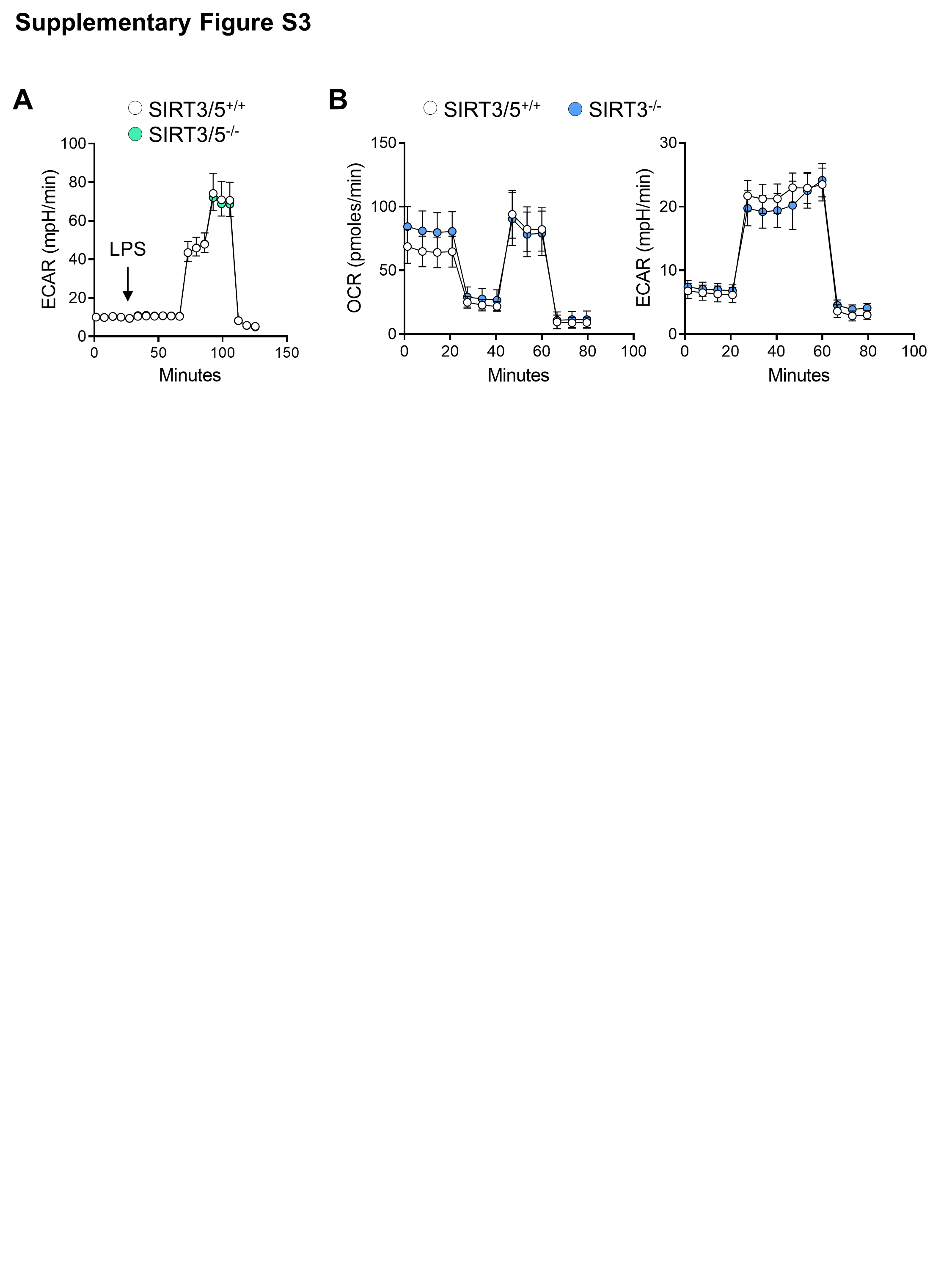

Supplement: Supplementary Figure S1 — Full blots used to extract the panels shown in Figures 1B, 3B. [file Presentation_1.zip › Image 3.TIF]
