# Supplementary material for: Impact of the Dual Deletion of the Mitochondrial Sirtuins SIRT3 and SIRT5 on Anti-microbial Host Defenses
Source: Front Immunol. 2019 Oct 1;10:2341. doi: 10.3389/fimmu.2019.02341 (PMC6781768; doi:10.3389/fimmu.2019.02341)
Supplement: Supplementary file 2 [file Data_Sheet_1.pdf]

Supplementary Information

1. Reagents

Reagent	Source	Identifier
Brain Heart Infusion	Oxoid	CM1135
Columbia III Agar with 5% Sheep Blood	BD	254098
CpG ODN: G*GGTCAACGTTGAG*G*G*G*G	Microsynth	
FBS Superior	Biochrom AG (Merck)	S 0615
HBSS no phenol red no Ca+ no Mg+	ThermoFisher	88284
IL-1 beta Mouse Uncoated ELISA kit	eBioscience	88-7013-88
IMDM, GlutaMAX	Gibco (ThermoFisher)	31980-022
KAPA SYBR Green Fast ROX low	Kapa Biosystems	KK4620
LPS ultrapure from *Salmonella minnesota*	List Biologicals	434
LS columns	Miltenyi	130-042-401
MitoSOX	ThermoFisher	M36008
Monosodium urate crystals	Sigma	21279999
Mouse Custom ProcartaPlex 17-plex	Invitrogen	PPX-17-MX7DPRR
Mouse Direct PCR Kit	Bimake	B4001
Mouse IL-10 ELISA development kit	Mabtech	3432-1H-20
Mouse IL-6 DuoSet ELISA	R&D Systems	DY406
Mouse TNF DuoSet ELISA	R&D Systems	DY410
Neutrophil isolation kit (mouse)	Miltenyi	130-097-658
Pam_3CSK_4	EMC microcollections	L2000
Penicillin-streptomycin	Gibco (ThermoFisher)	15140-122
Phorbol 12-myristate 13-acetate (PMA)	Enzo Life Sciences	BML-PE160-0001
Poly(I:C)	Invivogen	tlrl-pic-5
QuantiTect reverse transcription kit	Qiagen	205313
Recombinant Mouse M-CSF	ImmunoTools	12343115
RNeasy kit	Qiagen	74106
RPMI 1640 Medium, GlutaMAX	Gibco (ThermoFisher)	61870-010
SYTOX™ Green Nucleic Acid Stain	Invitrogen	S7020
Western Bright ECL HPR substrate	Advansta	K-12045-D20
XF Cell Mito Stress Test Kit	Agilent	103015-100
XF Glycolysis Stress Test Kit	Agilent	103020-100
XF Mito Fuel Flex Test Kit	Agilent	103260-100

2. Mouse strains

Name	Source	Identifier
C57BL/6J mice	Charles River	632
$SIRT3^{-/-}$ C57BL/6J mice	PMID: 22645641	-
$SIRT5^{-/-}$ C57BL/6J mice	PMID: 24076663	-
$SIRT3/5^{-/-}$ C57BL/6J mice	This paper	-

3. Oligonucleotides used for genotyping

Target	Primer number	Sequence (5'-3')
Lox2	903	GCTATGTAAGCACACAAGCTCACCTT
Sirt3	947	GATTCCTGATGCAAGATGGTTCTGTGC
Sirt3	948	CAGTCTTAGGCTAGCAAGAGTGAGG
Sirt5	2470	GTGTATAGTTGTGTGCTGTGTGCTTGTAC

Sirt5	2471	GGGAAAGATCTGGGGTTGGAATTTACC
Lox2	2468	CTTGAGCAGAAAACCACAGAGGAGAGAAC

4. Oligonucleotides used for RT-PCR

Target	Forward (5'-3')	Reverse (3'-5')
Acadl	CCACACAGAATGGGAGAAAGCT	AGAGCAAGTCCCCACCAATG
Cpt1	CAAACCTATTCGTCTTCTGGGATCT	TGAAGAGTCGCTCCCACTGA
Fabp4	TGGGAACCTGGAAGCTTGTC	GCAAAGCCCACTCCCACTT
Hadha	TCAGTCGCTTCTCTGCCTTCA	CAGCAGAGCAGAAGACGTTGTAA
Hmgcr	CCAAACCCCGTAACCCAAA	CGACTATGAGCGTGAACAAGGA
Idh1	TGTCCAGATGGTAAGACGGTAGAA	GGCAAAAATGGAAGCAATGG
Idh2	CAACACCGACGAGTCCATTTC	CTCAAGTAGAGCGGCCATTTCT
Mrpl19	GCCCAAGCCGATTTCAGA	AATTCAGGACTCAGGAACCTTCTC
Mvd	GACCAGCTAAAAACGACCACAAC	CCTCCTCGCGACCATTCA
Sod1	CCAGTGCAGGACCTCATTTTAAT	TCTCCAACATGCCTCTCTTCATC
Sod2	CTCTGGCCAAGGGAGATGTTAC	ATATGTCCCCCACCATTGAACT
Sqle	GGAAGAGCCTCATCTCCAGTAAAG	CTGTGGTGCATCCTTCATAAGG

5. Microorganisms

Species	Strain	Reference
L. monocytogenes	10403s	NCBI:txid393133

6. Antibodies used for Western blotting

Target	Source	Brand	Reference
Acetylated α tubulin	Mouse	Santa-Cruz	sc-23950
Acetyl-lysine	Mouse	Merck	05-515
β-actin	Rabbit	Cell Signaling	4967
NF-κB p65	Rabbit	Cell Signaling	8242
p38 MAPK	Rabbit	Cell Signaling	9212
p44/42 MAPK (Erk1/2)	Rabbit	Cell Signaling	9102
Phospho-p38 MAPK (Thr180/Tyr182)	Rabbit	Cell Signaling	9211
Phospho-p44/42 MAPK (Erk1/2 (Thr202/Tyr204)	Rabbit	Cell Signaling	9101
SIRT3	Rabbit	Cell Signaling	5490
SIRT5	Rabbit	Cell Signaling	8782
Tubulin	Mouse	Sigma-Aldrich	T5168
Rabbit IgG	Goat	Invitrogen	31460
Mouse IgG	Goat	Invitrogen	31430

7. Antibodies used for flow cytometry

Target	Clone name	Coupling	Brand	Reference
**Analysis of bone marrow**				
CD117 (c-kit)	2B8	APC	BioLegend	105812
Sca-1 (Ly-6A/E)	D7	FITC	eBioscience	11-5981-82
CD48	HM48-1	APC-Cy7	BioLegend	103432
CD150	TC15-12F12.2	PerCP-Cy5.5	BioLegend	115922
CD135	A2F10	PE	eBioscience	12-1351-82
CD41	MWReg30	eFluor450	eBioscience	48-0411-82

Sca-1	D7	Super Bright 600	eBioscience	63-5981-80
CD16/32	93	Alexa Fluor 700	eBioscience	56-0161-82
CD34	RAM34	FITC	eBioscience	11-0341-82
CD3e	17A2	PE-Cy7	BioLegend	100220
CD11b	M1/70	PE-Cy7	BioLegend	101216
Ly6C/G (Gr-1)	RB6-8C5	PE-Cy7	BioLegend	108416
Ly-76	TER119	PE-Cy7	BioLegend	116222
B220 (CD45R)	RA3-6B2	PE-Cy7	BioLegend	103222
**Analysis of spleen and thymus**				
B220	RA-6B2	Brilliant Violet 570	BioLegend	103237
IgD	11-26c	PE	eBioscience	12-5993-82
CD23	B3B4	Alexa Fluor 700	BioLegend	101631
CD11c	N418	APC	BioLegend	117310
CD8a	53-6.7	FITC	BioLegend	100706
CD4	GK1.5	APC-Cy7	BioLegend	100414
CD44	IM7	eFluor450	eBioscience	48-0441-82
CD62L	MEL-14	PerCP-Cy5.5	BD Biosciences	560513
CD11c	N418	PE	BioLegend	117307
CD11b	M1/70	PercCP-Cy5.5	eBioscience	45-0112-82
Ly6C	HK1.4	APC	BioLegend	128016
CD25	PC61	PerCP-Cy5.5	BD Biosciences	551071
**Live/dead differentiation**				
Zombie			BioLegend	77168
Fixable Aqua			ThermoFisher	L34957
Fixable Violet			ThermoFisher	L34955
